# A case of combined pulmonary vein isolation (PVI) and watchman implant through hepatic vein in a patient with interrupted inferior vena cava (IVC)

**DOI:** 10.1002/ccr3.7787

**Published:** 2023-08-10

**Authors:** Sameh Girgis, Negar Niknam, Zabeer Bhatti, Jalal Mohsin, Ahmed Kamel Abdel Aal, Ramesh Hariharan, Khashayar Hematpour

**Affiliations:** ^1^ Electrophysiology Department The University of Texas Health Science Center Houston Texas USA; ^2^ Internal Medicine Department University of Houston, HCA Kingwood Hospital Houston Texas USA; ^3^ University of Houston Houston Texas USA; ^4^ Diagnostic and Interventional Imaging The University of Texas Health Science Center Houston Texas USA

**Keywords:** atrial fibrillation, interrupted inferior vena cava, left atrial ablation, pulmonary vein isolation, transhepatic access, watchman device

## Abstract

This case report describes a successful procedure involving pulmonary vein isolation (PVI) and left atrial appendage (LAA) closure with a watchman device in a 78‐year‐old male with atrial fibrillation and an interrupted inferior vena cava. Due to the vascular anomaly, a transhepatic approach was used, which proved successful.

## 1 INTRODUCTION

There are scenarios where accessing the right atrium through cannulating the femoral veins is not feasible. These instances include chronic venous occlusion, interrupted inferior vena cava (IVC), and venous heterotaxia syndrome, a rare congenital disease with the abnormal arrangement of thoracoabdominal organs across the left to the right axis.^1^

We reported a case series of three patients previously who had interrupted IVC and required left atrial ablation. We performed the ablations successfully through hepatic vein cannulation and reported the characteristics of that approach.^2^ There are few case reports on watchman implantation through hepatic vein cannulation in the literature.^3^, ^4^, ^5^ This case report, to our knowledge, is the first reported combined PVI and LAA closure using a watchman device in a patient through hepatic vein cannulation.

A 78‐year‐old Caucasian male with a known history of symptomatic paroxysmal atrial fibrillation (afib) and New York Heart Association (NYHA) class II congestive heart failure presented with exertional palpitations and associated fatigue. He had documented history of paroxysmal afib for 2 years with progression to persistent afib in the preceding 3 months. Amiodarone and direct current cardioversion (DCCV) twice failed to maintain sinus rhythm. The patient was therefore referred for pulmonary vein isolation (PVI). He was also a candidate for LAA closure due to CHA_2_DS_2_‐VASc* 1of 5 and history of gastrointestinal (GI) bleeds. PVI was attempted but was aborted due to a total interruption of the IVC, leaving no venous access to the heart except through hepatic vein access.

**FIGURE 1:**
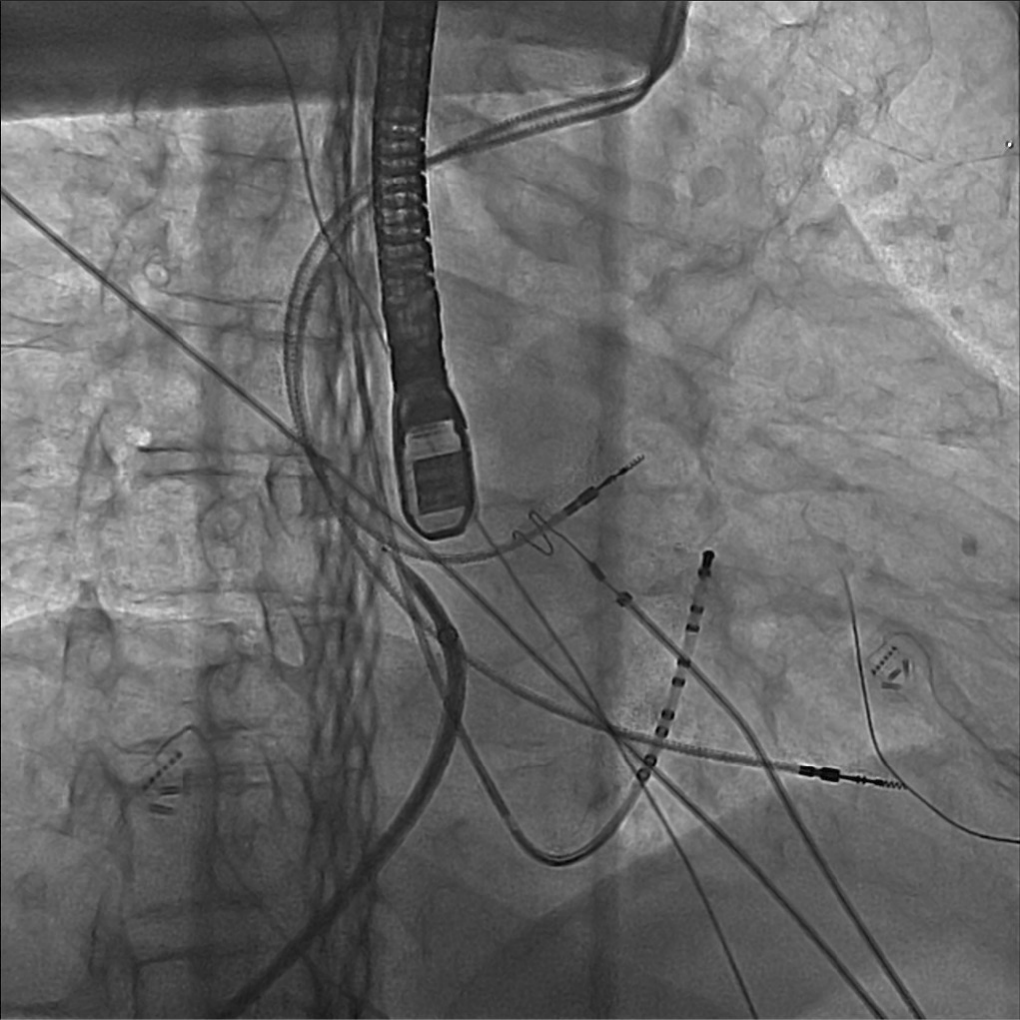
An almost horizontal and significant anterior projectory of the SL1 sheath placed in the right atrium necessitates posteriorly rotating the sheath significantly beyond the level that is required during the conventional approach.

Due to recurrent symptoms and inability to tolerate anticoagulation, it was decided to proceed with trans‐hepatic access for atrial fibrillation ablation and implantation of the watchman device simultaneously. Right internal jugular (IJ) access was obtained under ultrasound guidance. The coronary sinus (CS) catheter was inserted through the right IJ access and placed into the CS under fluoroscopy. Transhepatic venous access was obtained by interventional radiology (IR) under fluoroscopy as shown in the images. A 22‐gauge Chiba needle was used to target the right hepatic vein, and a 0.014‐inch wire was negotiated through the right hepatic vein into the IVC. A NEFF set (Cook) was then introduced through which the Swartz Braided SL Trans‐septal Guiding Introducer Sheath (SL1) guidewire was negotiated through the IVC and right atrium (RA) with its tip placed in the superior vena cava (SVC). SL1 sheath was then advanced over the guidewire that was placed by IR into the SVC. Transesophageal echocardiography (TEE) was used for guidance of transseptal access. A single transseptal puncture was performed after adequate anticoagulation with intravenous heparin. An almost horizontal and significant anterior projectory of the SL1 sheath placed in the right atrium necessitates rotating the sheath posteriorly, significantly beyond the level required during the conventional approach (Figure 1). Transseptal access was completed by directing the needle and sheath toward the right upper pulmonary vein as the anterior direction of the sheath warranted as such. SL1 was then exchanged for an Agilis NxT Steerable Introducer (Agilis sheath). PVI was done in a standard fashion using a single transseptal access and a TactiCath Quartz Contact Force ablation catheter by Abbott biomedical. Posterior wall isolation and mitral isthmus ablation lines were also performed and completed. Bidirectional exit block in the veins, posterior wall and mitral isthmus lines were confirmed. As we reported previously the right lower pulmonary vein (RLPV) was the most difficult to reach. In the process of ablating the RLPV the sheath and catheter did flip to the right atrium. The 3D mapping was used to guide the ablation catheter and then the agilis sheath back to the left atrium.

**FIGURE 2:**
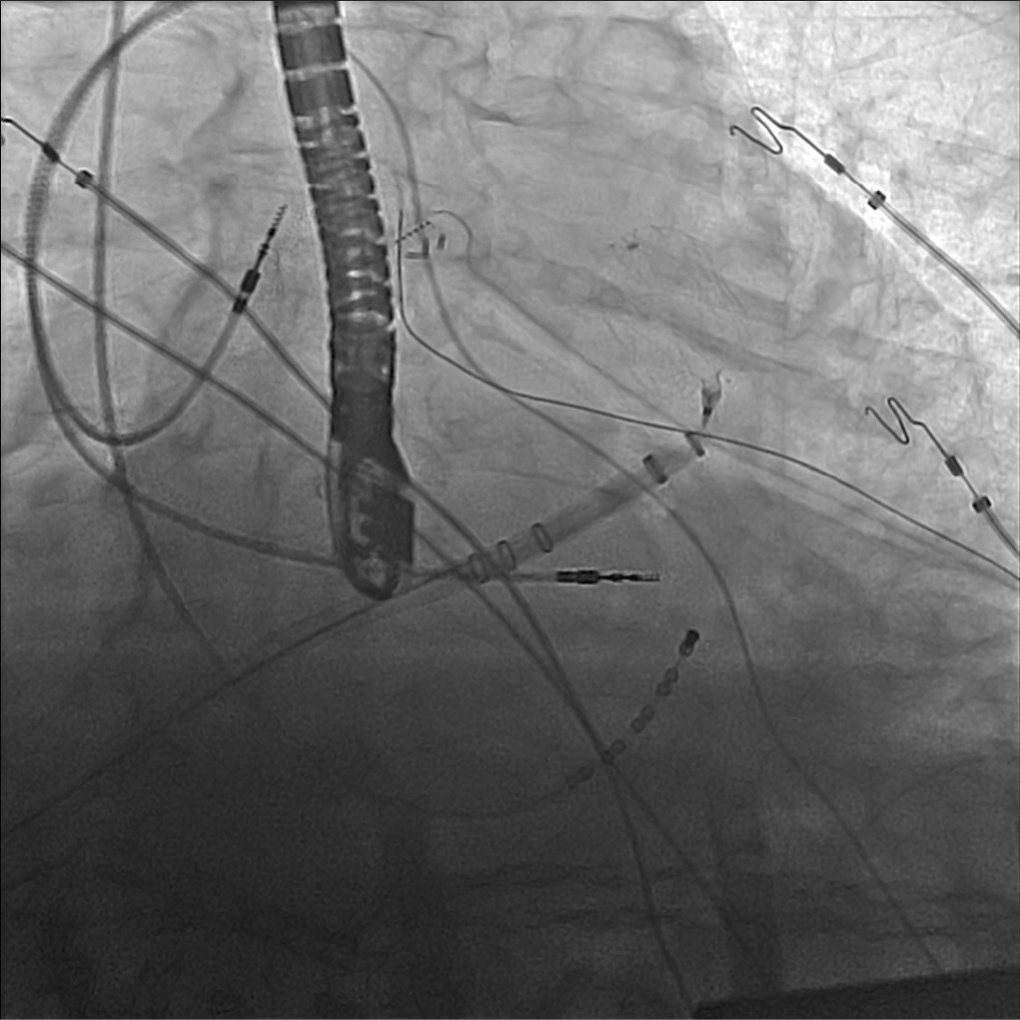
Deployment of the watchman device.

After completion of the PVI and the lines, an Amplatz Super Stiff™ Guidewire was advanced through the sheath into the left upper pulmonary vein. The agilis sheath was exchanged for a double‐curve watchman delivery sheath. Due to the inability to guide the sheath into the LAA over a pigtail catheter, the Advisor™ HD Grid Mapping Catheter originally used for mapping was used again to guide the sheath into the left atrial appendage which had a chicken wing morphology with a dominant anterior wing. Watchman device implant was done in the standard fashion. We felt the projection of the delivery sheath was not impeding the delivery of the closure device, and the overall angle was friendly for the watchman delivery (Figure 2).

**FIGURE 3:**
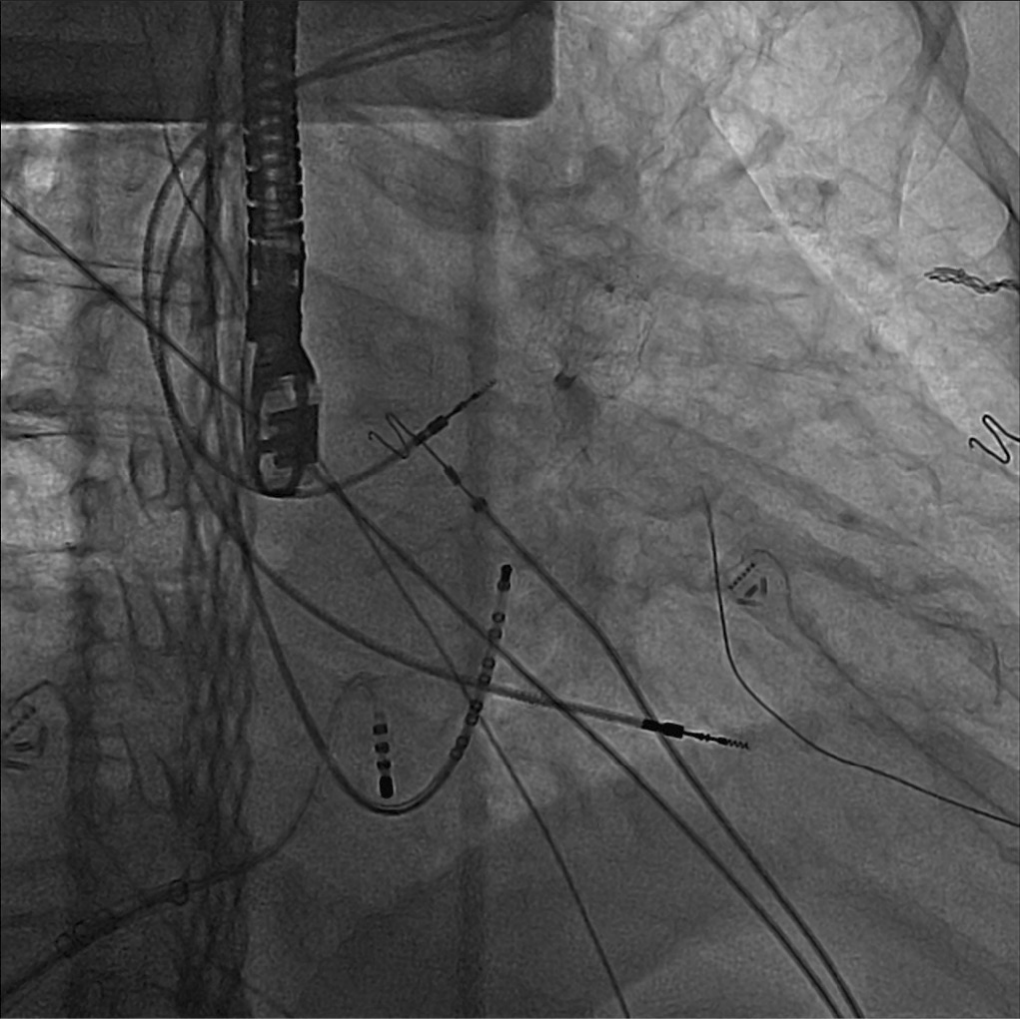
Placing the sheath on the CTI line.

The watchman sheath was then pulled back to the right atrium. The cavo‐tricuspid isthmus (CTI) was mapped, and CTI ablation was done in a standard fashion. Due to the almost 90‐degree angle between the catheter entrance projection and the CTI line, we had to use an exaggerated bend in the ablation catheter (Figure 3).

The transhepatic access was then closed by IR using two 8 mm Amplatzer Vascular Plugs type 4 (Abbott) which were deployed in the access tract through the access sheath, followed by Gelfoam injection under fluoroscopic guidance. Post‐embolization contrast injection revealed successful embolization of the tract through coils placement and injecting foam into the transhepatic access for hemostasis. The 14‐French venous sheath was then removed completely. The patient tolerated the procedure well and was discharged home after 3 days to allow for dofetilide loading without any complications.

## 2 DISCUSSION

Here we presented a PVI + Watchman implantation case with interrupted IVC, and we proceeded through transhepatic access to overcome this particular challenge. This has been described before for ablation purposes, but we here discuss using it for occlusion device placement and elaborate on some techniques that could help these patients. The first challenge in the case was proceeding with the transseptal access due to the difficult and unusual alignment of the transseptal access system and the usual human atrial septal anatomy. In this situation, after the initial septostomy with the needle and the dilator, pointing the dilator toward the right upper pulmonary vein can help pass the sheath through the transseptal access. The second important challenge in the case was the difficulty in the positioning of the watchman delivery system into the left atrial appendage. Here we described the use of the mapping catheter as a steerable body to guide the sheath into the left atrial appendage. After placing the sheath inside the LAA, the delivery of the watchman device was uneventful, possibly due to the dominant anterior wing, which was aligned with the projectory of the sheath.

Based on our experience, we recommend using a short deflectable agilis or any other 8.5 French deflectable sheath when using this approach due to the proximity of transhepatic access to the point of entry of the catheter to the skin compared to the groin access. This will help leave less excessive length of the sheath outside the skin entrance and offers better maneuverability. In addition, for our next case, we may ablate the CTI line using a Swartz Right (SR) 3 or 4 prior to septostomy. As shown in (Figure 3), placing a sheath in the CTI line would be easier and safer using a sheath that offers steep rightward access.

Performing afib ablation combined with the placement of the Watchman device for LAA occlusion has been evaluated before, with results showing evidence of safety and ability to perform.^6^ This can save time and materials allowing patients to have two procedures simultaneously, preventing repeating exposure to possible risks that come with general anesthesia and transseptal puncture, and decreasing the total length of hospital stay.^7^, ^8^ In this case, especially we proceeded with the combined procedure due to difficulty in obtaining access associated with total interruption of IVC and the obligation to use alternative nonconventional ways as the transhepatic access that may lead to higher risk upon repeating.

## AUTHOR CONTRIBUTIONS

**Sameh Girgis:** Writing – original draft. **Negar Niknam:** Writing – review and editing. **Zabeer Bhatti:** Writing – original draft. **Jalal Mohsin:** Data curation. **Ahmed Kamel Abdel Aal:** Resources; supervision. **Ramesh Hariharan:** Resources; supervision. **Khashayar Hematpour:** Project administration; supervision; writing – review and editing.

## FUNDING INFORMATION

None.

## CONFLICT OF INTEREST STATEMENT

All authors declare that they have no conflicts of interest.

## CONSENT

Written informed consent was obtained from patients to publish this report in accordance with the journal's patient consent policy.

## Data Availability

The data used to support the findings of this study are available upon request from the corresponding author, in accordance with data privacy and protection regulations.

## Footnotes

* CHA_2_DS_2_‐VASc score: congestive heart failure, hypertension, age, diabetes mellitus, prior stroke or TIA or thromboembolism, vascular disease, age, sex category.
